# Fibroblast Growth Factor 10-Fibroblast Growth Factor Receptor 2b Mediated Signaling Is Not Required for Adult Glandular Stomach Homeostasis

**DOI:** 10.1371/journal.pone.0049127

**Published:** 2012-11-01

**Authors:** Allison L. Speer, Denise Al Alam, Frederic G. Sala, Henri R. Ford, Saverio Bellusci, Tracy C. Grikscheit

**Affiliations:** 1 Children's Hospital Los Angeles, Department of Pediatric Surgery/Developmental Biology and Regenerative Medicine, Los Angeles, California, United States of America; 2 University of Giessen Lung Center, Department of Internal Medicine II, Giessen, Germany; Oregon Health & Science University, United States of America

## Abstract

The signaling pathways that are essential for gastric organogenesis have been studied in some detail; however, those that regulate the maintenance of the gastric epithelium during adult homeostasis remain unclear. In this study, we investigated the role of Fibroblast growth factor 10 (FGF10) and its main receptor, Fibroblast growth factor receptor 2b (FGFR2b), in adult glandular stomach homeostasis. We first showed that mouse adult glandular stomach expressed *Fgf10*, its receptors, *Fgfr1b* and *Fgfr2b*, and most of the other FGFR2b ligands (*Fgf1, Fgf7, Fgf22*) except for *Fgf3* and *Fgf20*. *Fgf10* expression was mesenchymal whereas FGFR1 and FGFR2 expression were mostly epithelial. Studying double transgenic mice that allow inducible overexpression of *Fgf10* in adult mice, we showed that *Fgf10* overexpression in normal adult glandular stomach increased epithelial proliferation, drove mucous neck cell differentiation, and reduced parietal and chief cell differentiation. Although a similar phenotype can be associated with the development of metaplasia, we found that *Fgf10* overexpression for a short duration does not cause metaplasia. Finally, investigating double transgenic mice that allow the expression of a soluble form of *Fgfr2b,* FGF10's main receptor, which acts as a dominant negative, we found no significant changes in gastric epithelial proliferation or differentiation in the mutants. Our work provides evidence, for the first time, that the FGF10-FGFR2b signaling pathway is not required for epithelial proliferation and differentiation during adult glandular stomach homeostasis.

## Introduction

Gastric adenocarcinoma is the fourth most common cancer and the second leading cause of cancer-related death worldwide [1] with an overall 5-year relative survival rate in most countries around 20% [2]. Gastric carcinogenesis is most commonly associated with H. Pylori infection, but other risk factors include nutritional consumption (high salt intake and/or low fruit and vegetable intake) as well as African-American ethnicity and low socioeconomic status [3]. Parietal cell loss, or oxyntic atrophy, is the most reliable preneoplastic correlate in humans. The loss of parietal cells, regardless of the cause (Helicobacter infection or pharmacologic agents), leads to the subsequent development of metaplasia and can be accelerated by gastrin or histamine deficiency [4], [5]. In humans, two types of mucous cell metaplasia can arise as a result of oxyntic atrophy: goblet cell intestinal metaplasia (IM) or spasmolytic polypeptide-expressing metaplasia (SPEM) [4], [6]. The Fibroblast growth factor (FGF), Hedgehog, Transforming growth factor beta (TGFβ)/Bone morphogenetic protein (BMP), and Wnt signaling pathways are important and related morphogenetic networks that regulate stem cells, particularly in the gastrointestinal tract [7], [8]. These signaling pathways are crucial during embryonic development, adult homeostasis, tissue repair and regeneration, and carcinogenesis. Defining the role of the FGF10-FGFR2b signaling pathway during adult glandular stomach homeostasis is a first step to delineating the cellular mechanisms of gastric epithelial repair and regeneration after injury.

Fibroblast growth factors (FGFs) play key roles in cellular proliferation, differentiation, migration, and inflammation in several organs [8]. FGFs bind to one or more tyrosine kinase transmembrane FGF receptors (FGFRs) [9]. As with several other highly conserved signaling pathways, FGF signaling tends to occur in a paracrine fashion between the epithelium and mesenchyme, with the FGF ligand expressed in the tissue adjacent to its corresponding FGFR(s) [10]. For example, during gastric organogenesis, *Fgf10* is expressed in the mesenchyme, whereas its main receptor, *Fgfr2IIIb* (hereafter *Fgfr2b*), is expressed in the epithelium [11], [12], [13].

We have previously reported that FGF10-FGFR2b mediated signaling is essential for the organogenesis of the stomach [13], the duodenum [14], [15], the cecum [16], [17] and the colon [18], [19], [20] in the mouse. Colonic atresia was associated with a decrease in epithelial proliferation and increase in epithelial apoptosis in both *Fgf10^−/−^* and *Fgfr2b^−/−^* mice [19], [20]. In contrast to the mesenchyme, the differentiation of the colonic epithelium was unaffected in *Fgf10^−/−^* [20]. During embryonic stomach development, both *Fgf10* and *Fgfr2b* knockouts had impaired epithelial proliferation, but conversely demonstrated severe compromise of gastric epithelial differentiation with an absence of parietal cells and reduced number of chief cells [13]. Moreover, ectopic overexpression of *Fgf10* during stomach development also revealed major alterations in epithelial differentiation including a reduction in parietal and endocrine cells and an increase in chief cells [11]. Despite these initial observations in gastrointestinal development, the role of FGF10-FGFR2b signaling in the stomach during adult homeostasis has not yet been investigated.

In this study, we investigate the role of FGF10-FGFR2b signaling during adult glandular stomach homeostasis. We first demonstrated the presence of *Fgf10* and its receptors, *Fgfr1b* and *Fgfr2b*, in adult glandular stomach, along with all the genes encoding the other FGFR2b ligands (*Fgf-1, −3, −7, −20, −22*). Investigating double transgenic mice allowing *Fgf10* overexpression, we showed that *Fgf10* overexpression increases epithelial proliferation, drives mucous neck cell differentiation and reduces parietal and chief cell differentiation during adult glandular stomach homeostasis. Although the loss of parietal cells and increase in mucus-producing cells can be associated with the development of metaplasia, we did not identify any metaplasia in our *Fgf10* overexpressing mutant mice as demonstrated by immunostaining for two well-described markers of metaplasia: CDX2 (IM) [21] and HE4 (IM and SPEM) [6]. Finally, ubiquitous expression of a dominant-negative soluble form of *Fgfr2b,* FGF10's main receptor, revealed no significant changes in gastric epithelial proliferation or differentiation in the mutants. Thus, this study demonstrates that FGF10-FGFR2b signaling is not required for adult glandular stomach homeostasis.

## Results

### Expression of *Fgf10,* its receptors, *Fgfr1b and Fgfr2b*, and the other FGFR2b ligands in adult mouse glandular stomach

**Figure 1:**
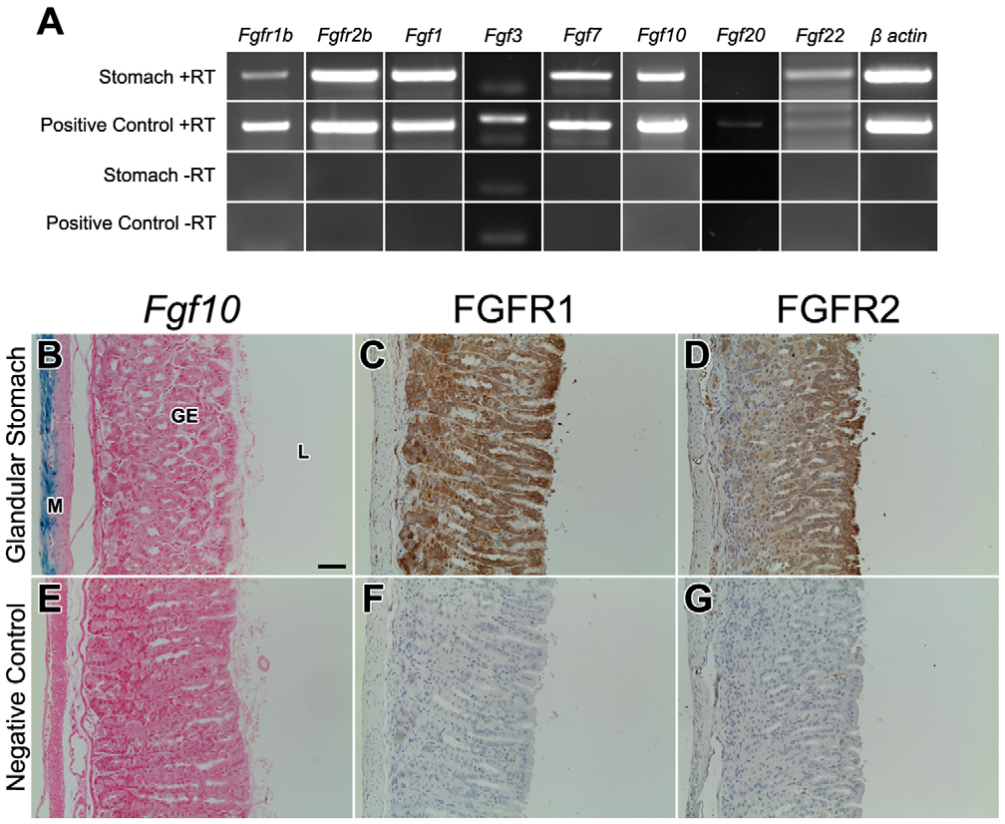
Normal expression pattern of *Fgf10,* its receptors, all other FGFR2b ligands in adult glandular stomach. (A) PCR of adult glandular stomach and E14.5 wildtype whole embryo (positive control). RT negative controls for both are all negative. (B,E) β-galactosidase staining of adult glandular stomach in *Fgf10^LacZ/+^* (B) and wildtype *Fgf10^+/+^* (E) mice. *Fgf10* expression occurs only in the mesenchyme. Immunohistochemical analysis of wildtype adult glandular stomach sections stained with anti-FGFR1 (C) or anti-FGFR2 (D). (F,G) Negative control stainings, in which the primary antibody was absent, show minimal to no signal. FGFR1 and FGFR2 staining is strong throughout the gastric epithelium whereas the mesenchymal staining is much weaker. The scale bar is 50 µm. M = mesenchyme, GE = gastric epithelium, L = lumen.

In order to study the expression of *Fgf10,* its receptors, *Fgfr1b* and *Fgfr2b*, and the other FGFR2b ligands in adult glandular stomach, we performed RT-PCR on wild type adult mouse glandular stomach (n = 3). Both receptors were expressed in adult glandular stomach and in the positive control, wild type E14.5 mouse whole embryo (Figure 1A). The genes encoding all of the ligands binding FGFR2b (*Fgf1*, *Fgf7*, *Fgf10*, *Fgf22*) were expressed in adult glandular stomach except for *Fgf3* and *Fgf20*, whereas as all six were expressed in the positive control (Figure 1A). RT negative controls for both adult glandular stomach and the positive control were negative for *Fgfr1b, Fgfr2b,* all FGFR2b ligands, and *β actin* (Figure 1A).

To determine the spatial expression pattern of *Fgf10*, we performed β-galactosidase staining on sections of adult glandular stomach from *Fgf10^LacZ/+^* reporter mice (n = 3), which have been previously validated [20], [22]. We found that *Fgf10* expression was present in the mesenchyme just beneath the gastric glands of the epithelium (Figure 1B). Negative controls (wild type *Fgf10^+/+^,* n = 3) showed no LacZ staining (Figure 1E). To confirm the expression of FGF10 receptors in adult stomach, we performed immunohistochemistry staining for FGFR1 and FGFR2 on wild type adult mouse glandular stomach (n = 3). Since specific antibodies for the IIIb isoform of these receptors are not available, antibodies that react with both the IIIb and IIIc isoforms of each receptor were used. The IIIb isoform is usually expressed in the epithelium whereas the IIIc isoform is typically expressed in the mesenchyme. Both FGFR1 and FGFR2 (Figure 1C and 1D, respectively) were identified with strong immunostaining in the gastric epithelium and weaker staining in the mesenchyme. Our negative controls did not show any specific staining (Figure 1F, 1G). The presence of *Fgf10* and its receptors in the adult mouse stomach suggests a role for FGF10 in stomach homeostasis. Therefore, we postulate that FGF10-FGFR2b signaling during adult glandular homeostasis, similar to previous studies, is likely a mesenchymal to epithelial signal.

### *Fgf10* overexpression during homeostasis changes gastric gland histology and increases epithelial proliferation in glandular stomach

**Figure 2:**
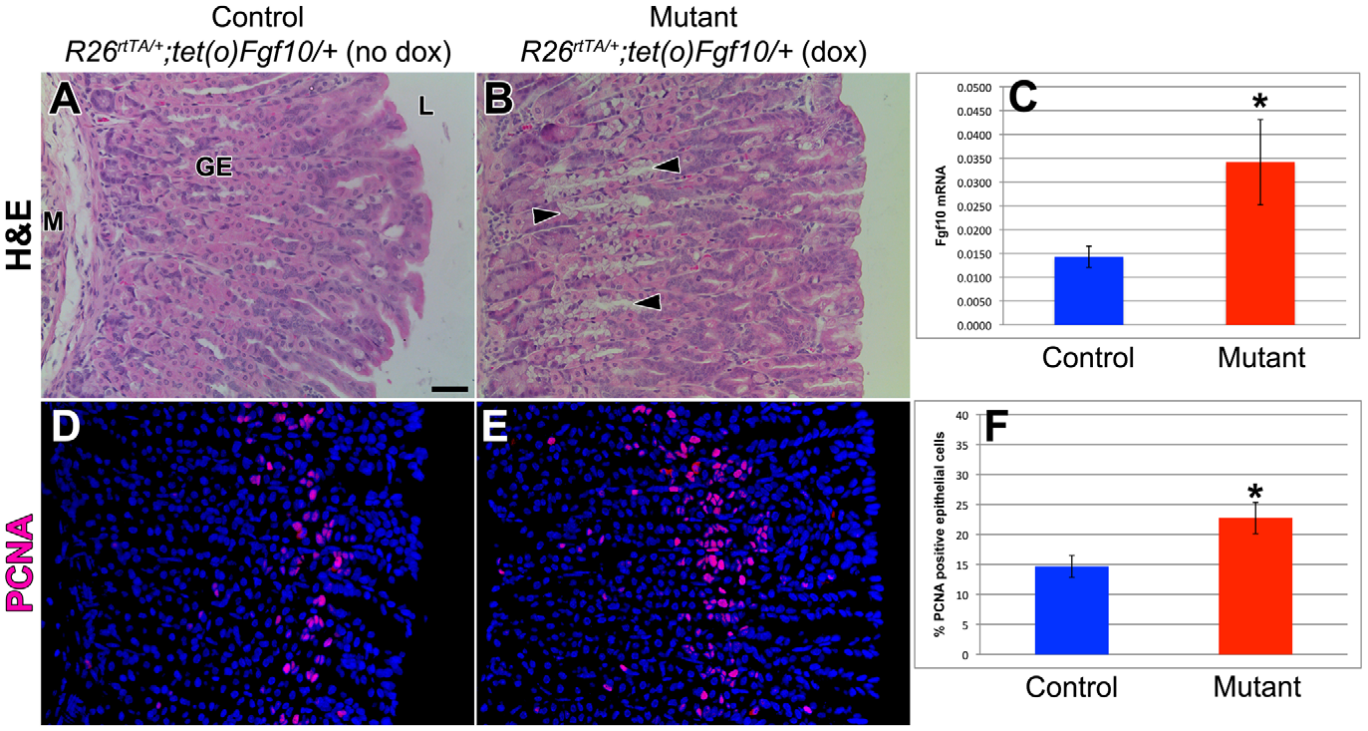
*Fgf10* overexpression during homeostasis changes gastric gland histology but not epithelial proliferation in glandular stomach. (A–B) Hematoxylin and eosin staining of sections of adult glandular stomach in control littermate (*R26^rtTA/+^; tet(O)Fgf10/+* without dox) (A) and mutant *Fgf10* overexpressing mice (*R26^rtTA/+^; tet(O)Fgf10/+* with dox) (B). There is a visible decrease in the number of parietal cells and an obvious shift of the mucous neck cells from the luminal side towards the gastric gland base (black arrowheads). (C) qRT-PCR showing a significant increase in Fgf10 expression in the mutants vs controls (*p<0.05). (D-E) PCNA immunostaining of sections of adult glandular stomach in control littermate (*R26^rtTA/+^; tet(O)Fgf10/+* without dox) (D) and mutant *Fgf10* overexpressing mice (*R26^rtTA/+^; tet(O)Fgf10/+* with dox) (E). (F) Quantification of the percentage of PCNA-positive epithelial cells showed a statistically significant increase in the percentage of PCNA positive epithelial cells indicating increased gastric epithelial proliferation in mutant *Fgf10* overexpressing mice (*R26^rtTA/+^; tet(O)Fgf10/+* with dox) compared to control littermates (*R26^rtTA/+^; tet(O)Fgf10/+* without dox). Dox was given for 10 days. The scale bar is 50 µm. M = mesenchyme, GE = gastric epithelium, L = lumen.

In order to investigate the role of FGF10 during adult glandular stomach homeostasis, we generated inducible double transgenic heterozygous mice that ubiquitously overexpressed *Fgf10* (*R26^rtTA/+^; tet(O)Fgf10/+* hereafter). Overexpression of *Fgf10* was induced by feeding doxycycline to adult (4 week old) mutant mice and control littermates for 10 days prior to sacrifice. The overexpression of *Fgf10* was confirmed by qRT-PCR and the mutants showed a significant increase in the expression of *Fgf10* when compared to control littermates (Figure 2C). Mutant mice typically develop a wet-hair appearance and an obvious proliferation of cutaneous epithelium, including swelling of the eyelids and an abnormally enlarged tongue.

Hematoxylin and eosin staining of sections of control littermate adult glandular stomach showed a simple columnar epithelium organized into gastric glands containing numerous parietal cells with large eosinophilic cytoplasm, chief cells at the base of the glands with basophilic cytoplasm, basally located nuclei, and apical secretory granules, and mucous neck cells with mucus seen in white at the apical portion of the cells in the neck of the glands (Figure 2A). This histology was significantly altered in the adult glandular stomach of the *Fgf10* overexpressing mutant mice (Figure 2B). There was a visible decrease in the parietal cell population, with an increase in mucus-secreting cells and a clustering of these cells closer to the base of the gland (black arrowheads).

As it is well established that FGF10 promotes proliferation in a number of organs including the gastrointestinal tract [13], [19], [20], [23], [24], we analyzed the proliferation of the gastric epithelium by PCNA immunostaining in the control littermates and mutant mice. Compared to littermate controls, the mutant glandular stomachs showed a significant increase in the proliferation of the epithelium (14.7±1.8% PCNA-positive epithelial cells vs. 22.7±2.6% in the mutants, p = 0.017, n = 5 for each genotype) (Figure 2D-F). Our data demonstrated that overexpression of FGF10 promotes cell proliferation in the epithelium of adult mouse glandular stomach.

### Gastric epithelial differentiation is significantly altered by *Fgf10* overexpression during homeostasis

**Figure 3:**
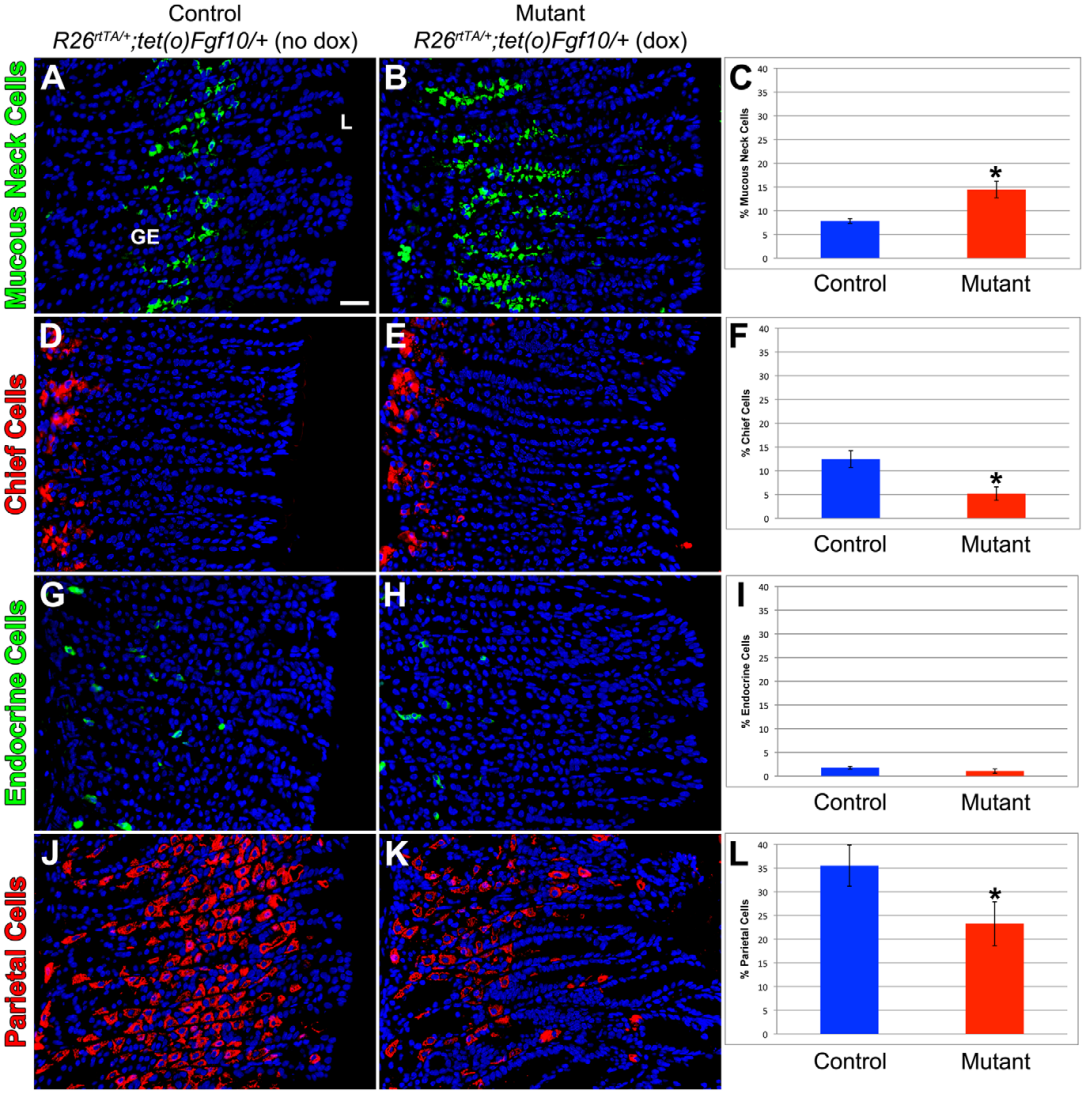
Gastric epithelial differentiation is significantly altered by *Fgf10* overexpression during homeostasis. Immunostaining for markers of terminally differentiated gastric epithelial cells of sections of adult glandular stomach in control littermate (*R26^rtTA/+^; tet(O)Fgf10/+* without dox) (A,D,G,J) and mutant *Fgf10* overexpressing mice (*R26^rtTA/+^; tet(O)Fgf10/+* with dox) (B,E,H,K). (A-B) Mucous neck cells are identified by GSII and (C) quantification reveals a significant increase in mutant *Fgf10* overexpressing mice compared to control littermates (*p<0.05). (D,E) Chief cells are marked by intrinsic factor and (F) quantification shows a significant decrease in chief cell number (*p<0.05). (G,H) Endocrine cells are identified by chromogranin A and (I) quantification demonstrates no significant change in endocrine cell number. (J,K) Parietal cells are visualized with H/K ATPase immunostaining and (L) quantification demonstrates a significant reduction in mutant *Fgf10* overexpressing mice compared to control littermates (*p<0.05). Dox was given for 10 days. The scale bar is 50 µm. GE = gastric epithelium, L = lumen.

In order to further define the role of FGF10 in epithelial differentiation during adult glandular stomach homeostasis, we performed immunostaining for differentiated gastric epithelial cell markers in mutant and control stomachs. Mucous neck cells in glandular stomach were identified by lectin GSI-II, which has been previously established as a mucous neck cell marker [25], [26], [27]. *Fgf10* overexpression resulted in an 85% increase in the percentage of mucous neck cells in the gastric epithelium of the mutant mice (Figure 3B) compared to control littermates (Figure 3A) (14.4±1.7% vs. 7.8±0.5%, p = 0.003, n = 5 for each genotype) (Figure 3C). Furthermore, the GS-II positive cells were located closer to the base of the glands in the mutant mice compared to the controls. This corroborates our histological data, and indicates that FGF10 may promote differentiation of the mucous neck cell lineage. Intrinsic factor (IF) immunostained the chief cells at the base of the gastric glands. Although IF is produced and released by parietal cells in humans, it is an established marker of chief cells in rodents [21], [28], [29]. Our results showed a significant decrease of chief cells in the gastric epithelium of the mutants (Figure 3E) compared to control littermates (Figure 3D) (5.2±1.4% vs. 12.4±1.7%, p = 0.006, n = 5 for each genotype) (Figure 3F). Chromogranin A is an acidic glycoprotein expressed in multiple types of neuroendocrine cells throughout the gastrointestinal tract including the stomach [11], [21], [30], [31]. In the stomach, chromogranin A marked a small number of epithelial cells scattered throughout the gastric glands. We found no significant difference in the percentage of endocrine cells in the gastric epithelium between the mutants (Figure 3H) and controls (Figure 3G) (1.0±0.5% vs. 1.7±0.3%, p = 0.11, n = 5 for each genotype) (Figure 3I). Figures 3J-K demonstrate H/K ATPase immunostaining of the parietal cells within the gastric gland. There is a noticeable decrease in the parietal cell lineage (35% reduction) in the mutants (Figure 3K) compared to controls (Figure 3J), as shown in Figure 3L (23.2±4.6% vs. 35.5±4.3%, p = 0.044, n = 5 for each genotype). These data suggest that FGF10 plays a role in the differentiation of gastric epithelial mucous neck cell, chief cell, and parietal cell lineages during adult glandular stomach homeostasis. *Fgf10* overexpression not only significantly alters the number of these differentiated epithelial cell types as described above, but also changes the location of the mucous neck cells from the neck to the base of the gastric gland.

### *Fgf10* overexpression during homeostasis does not cause metaplasia of the gastric epithelium

**Figure 4:**
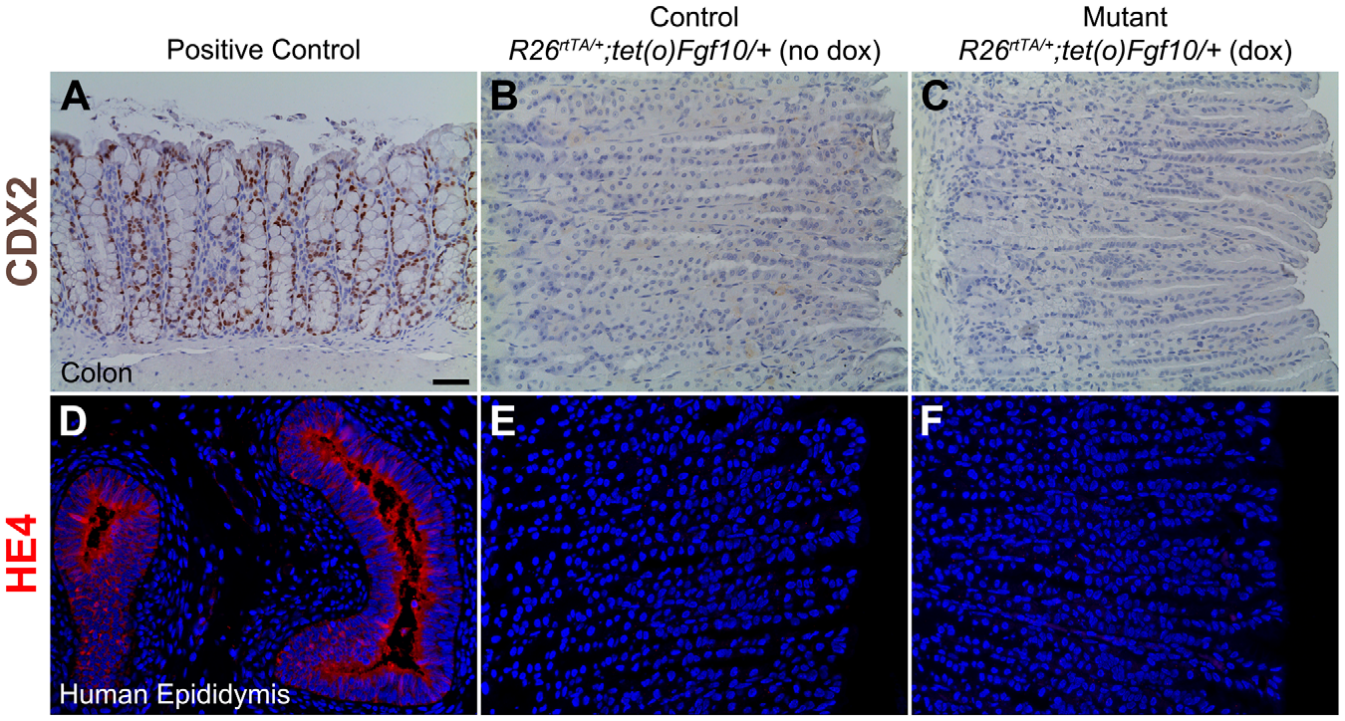
*Fgf10* overexpression during homeostasis does not cause metaplasia of the gastric epithelium. (A–C) CDX2, a marker of intestinal metaplasia (IM), immunostaining in adult colon (positive control) (A), control littermate (*R26^rtTA/+^; tet(O)Fgf10/+* without dox) (B) and mutant *Fgf10* overexpressing mice (*R26^rtTA/+^; tet(O)Fgf10/+* with dox) (C). (D-F) HE4, a marker of spasmolytic polypeptide expressing metaplasia (SPEM), immunostaining in human epididymis (positive control) (D), control littermate (*R26^rtTA/+^; tet(O)Fgf10/+* without dox) (E) and mutant *Fgf10* overexpressing mice (*R26^rtTA/+^; tet(O)Fgf10/+*) (F). Dox was given for 10 days. The scale bar is 50 µm.

Intestinal metaplasia (IM) and spasmolytic polypeptide-expressing metaplasia (SPEM) are both metaplasias of the gastric epithelium that typically develop after acute oxyntic atrophy and result in an increase in mucus-secreting cells: intestinal goblet cells in IM or mucous neck cells in SPEM [32]. It is currently thought that the loss of parietal cells results in transdifferentiation of mature chief cells in addition to inhibition of the normal mucous neck cell to chief cell differentiation [5], [21], [33]. Since the mutant mice demonstrated a similar phenotype with a significant loss of parietal cells, increase in mucous neck cells, and reduction in chief cells, we sought to investigate if *Fgf10* overexpression could cause metaplasia. In order to confirm if metaplasia was present in our mutant mice, we performed immunostaining for two well-established markers of metaplasia in both mice and humans: CDX2 (IM) [21] and HE4 (IM and SPEM) [6]. Both the mutant (Figure 4C) and the littermate control stomachs (Figure 4B) demonstrated no appreciable CDX2 staining in the gastric epithelium (n = 3 for each genotype). Colon served as a positive control and showed appropriate nuclear staining for CDX2 (Figure 4A). Similarly, no detectable HE4 staining was observed in the gastric epithelium of the mutants (Figure 4F) and littermate controls (Figure 4E) (n = 3 for each genotype). Human epididymis served as a positive control and had visible cytoplasmic staining for HE4 (Figure 4D). These results suggest that although *Fgf10* overexpression may result in a SPEM-like phenotype, the gastric epithelium is not metaplastic.

### FGF10-FGFR2b mediated signaling is not required for gastric epithelial proliferation and differentiation during homeostasis

**Figure 5:**
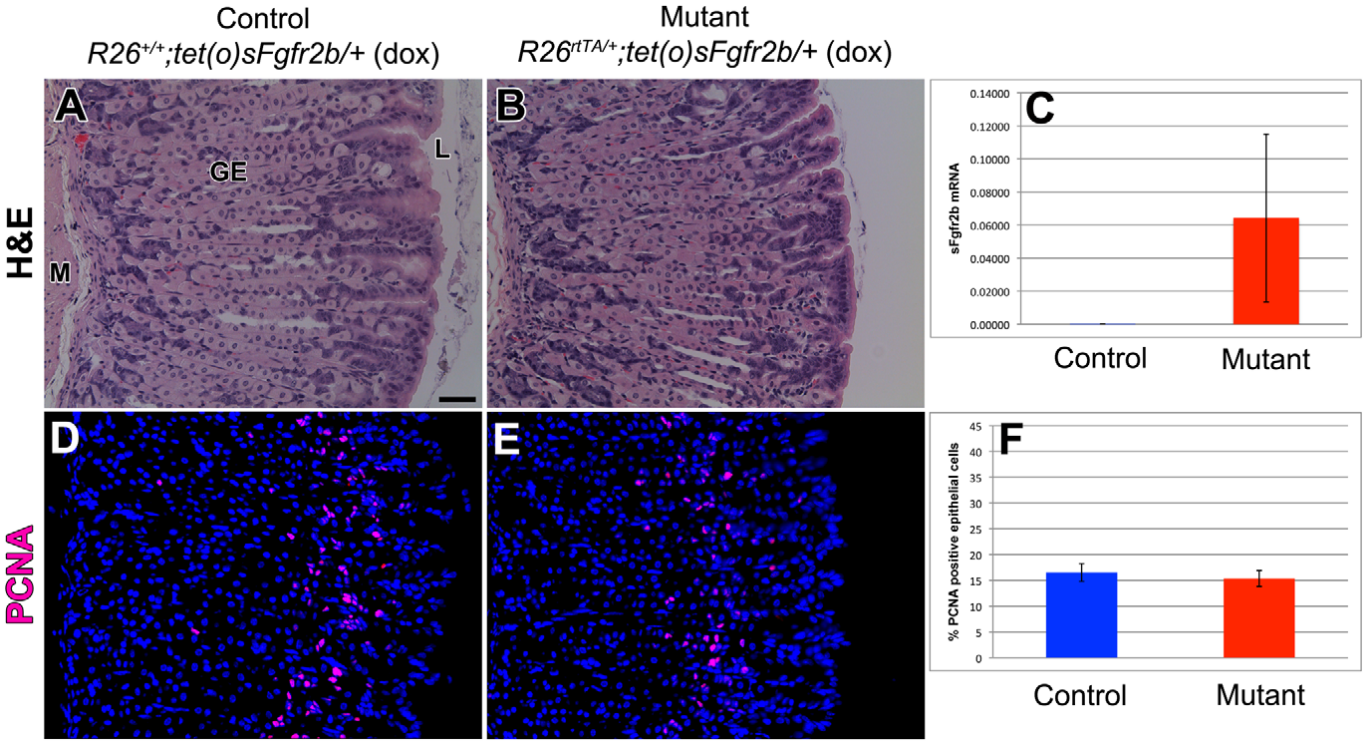
FGF10-FGFR2b mediated signaling is not required for normal gastric histology and epithelial proliferation during homeostasis. Hematoxylin and eosin staining of sections of adult glandular stomach in control littermate (*R26^+/+^; tet(O)sFgfr2b/+* with dox) (A) and mutant mice (*R26^rtTA/+^; tet(O)sFgfr2b/+* with dox) (B) showing normal stomach histology. (C) qRT-PCR confirming robust expression of *sFgfr2b* in the mutants as compared to controls where the level of *sFgfr2b* is nearly undetectable. PCNA immunostaining of sections of adult glandular stomach in control littermate (*R26^+/+^; tet(O)sFgfr2b/+* with dox) (D) and mutant mice (*R26^rtTA/+^; tet(O)sFgfr2b/+* with dox) (E) and (F) quantification of the percentage of PCNA-positive epithelial cells showing no significant change in gastric epithelial proliferation in mutant mice compared to control littermates. Dox was given for 1 month. The scale bar is 50 µm. M = mesenchyme, GE = gastric epithelium, L = lumen.

In order to determine the importance of the FGF10-FGFR2b signaling axis during adult glandular stomach homeostasis, we generated inducible double transgenic heterozygous mice that ubiquitously express a dominant-negative soluble form of *Fgfr2b* (*R26^rtTA/+^; tet(O)sFgfr2b/+* hereafter). Expression of *sFgfr2b* was induced by feeding doxycycline to adult (4 week old) mutant mice and control littermates for 1 month prior to sacrifice. The expression of *sFgfr2b* in the mutant mice was confirmed by qRT-PCR. The controls had nearly undetectable amounts of *sFgfr2b*, while the mutants had a variable but robust expression (Figure 5C). Expression of *sFgfr2b* acts in a dominant negative fashion by binding all FGFR2b ligands (FGFs-1, −3, −7, −10, −20, −22) and preventing their action. This has been previously validated in our laboratory where we demonstrated that inducible expression of *sFgfr2b* during embryonic development phenocopied *Fgfr2b^−/−^* embryos [34], whereas single transgenic embryos exposed to dox and double transgenic embryos not exposed to dox were identical to wild type embryos [35]. In contrast to the severe phenotype when FGFR2b is inactivated during embryogenesis, the postnatal expression of *sFgfr2b* results only in minor defects including defective incisors, longer claws, and reduced white adipose tissue [36].

Hematoxylin and eosin staining of sections of control littermate (Figure 5A) and mutant (Figure 5B) adult glandular stomachs revealed normal histology with appropriate gastric gland architecture and these were indistinguishable from one another. FGF10-FGFR2b mediated signaling has been shown to be required for epithelial proliferation during both gastric and colonic development [13], [19], [20]. In order to ascertain if FGF10-FGFR2b signaling is also necessary for gastric epithelial proliferation during homeostasis, immunostaining for PCNA was performed in the control littermate (Figure 5D) and mutant (Figure 5E) adult glandular stomachs. There was no difference in the rate of proliferation between the littermate controls and mutants (16.5±1.7% vs. 15.3±1.5%, p-value = 0.313, n = 5 for each genotype) (Figure 5F).

**Figure 6:**
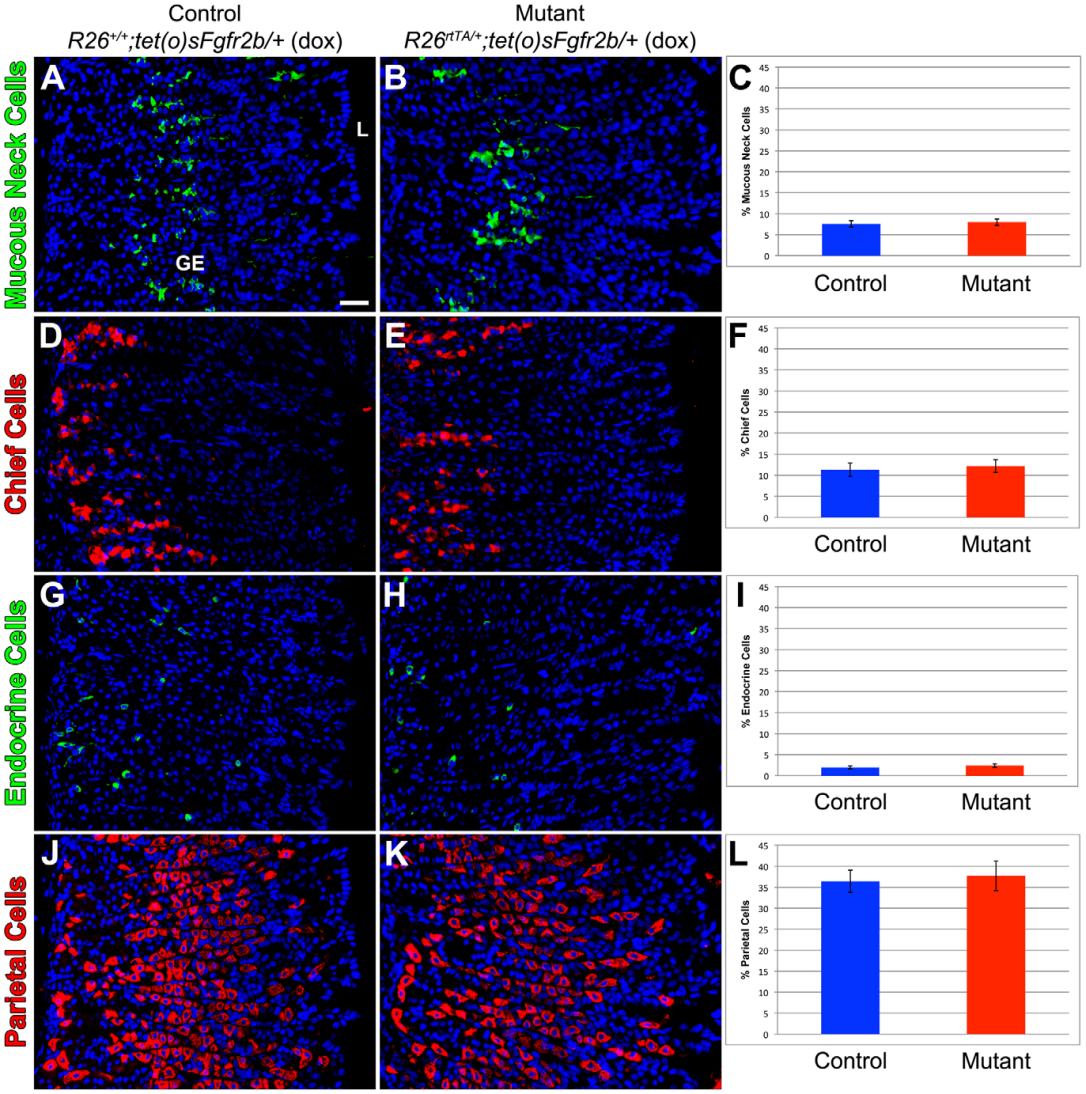
FGF10-FGFR2b mediated signaling is not required for gastric epithelial differentiation during homeostasis. Mucous neck cells are marked by GSII immunostaining of sections of adult glandular stomach in control littermate (*R26^+/+^; tet(O)sFgfr2b/+*with dox) (A) and mutant mice (*R26^rtTA/+^; tet(O)sFgfr2b/+* with dox) (B) and (C) quantification reveals no difference in mucous neck cell number. Chief cells are identified by intrinsic factor immunostaining of sections of adult glandular stomach in control littermate (*R26^+/+^; tet(O)sFgfr2b/+* with dox) (D) and mutant mice (*R26^rtTA/+^; tet(O)sFgfr2b/+* with dox) (E) and (F) quantification reveals no significant change in chief cell number. (G,H) Endocrine cells are identified by chromogranin A and (I) quantification shows no significant change in mutant *sFgfr2b* overexpressing mice compared to control littermates. Parietal cells are identified by H/K ATPase immunostaining of sections of adult glandular stomach in control littermate (*R26^+/+^; tet(O)sFgfr2b/+*with dox) (J) and mutant mice (*R26^rtTA/+^; tet(O)sFgfr2b/+* with dox) (K) and (L) quantification reveals no significant change in parietal cell number. Dox was given for 1 month. The scale bar is 50 µm. GE = gastric epithelium, L = lumen.

As it has been previously demonstrated that FGF10-FGFR2b signaling is essential for epithelial differentiation during gastric organogenesis, particularly for the development of the parietal cell lineage [13], we sought to determine if FGF10-FGFR2b mediated signaling was required for gastric epithelial differentiation during homeostasis. Sections of glandular stomach from the *R26^rtTA/+^; tet(O)sFgfr2b/+* mutant mice and control littermates were immunostained with markers for differentiated gastric epithelial cells. There was no difference in the percentage of mucous neck cells (7.5±0.8% vs. 8.0±0.8%, p = 0.35, n = 5 for each genotype) (Figures 6A–C), chief cells (11.3±1.6% vs. 12.2±1.5%, p = 0.35, n = 5 for each genotype) (Figures 6D–F), endocrine cells (1.9±0.3% vs. 2.4±0.4%, p = 0.22, n = 5 for each genotype) (Figures 6G–I), or parietal cells in the gastric epithelium (36.4±2.7% vs. 37.7±3.5%, p = 0.38, n = 5 for each genotype) (Figures 6J-K), between the littermate control and the mutant stomachs. Taken together, these results suggest that FGFR2b signaling is not required for gastric epithelial proliferation and differentiation during homeostasis.

**Figure 7:**
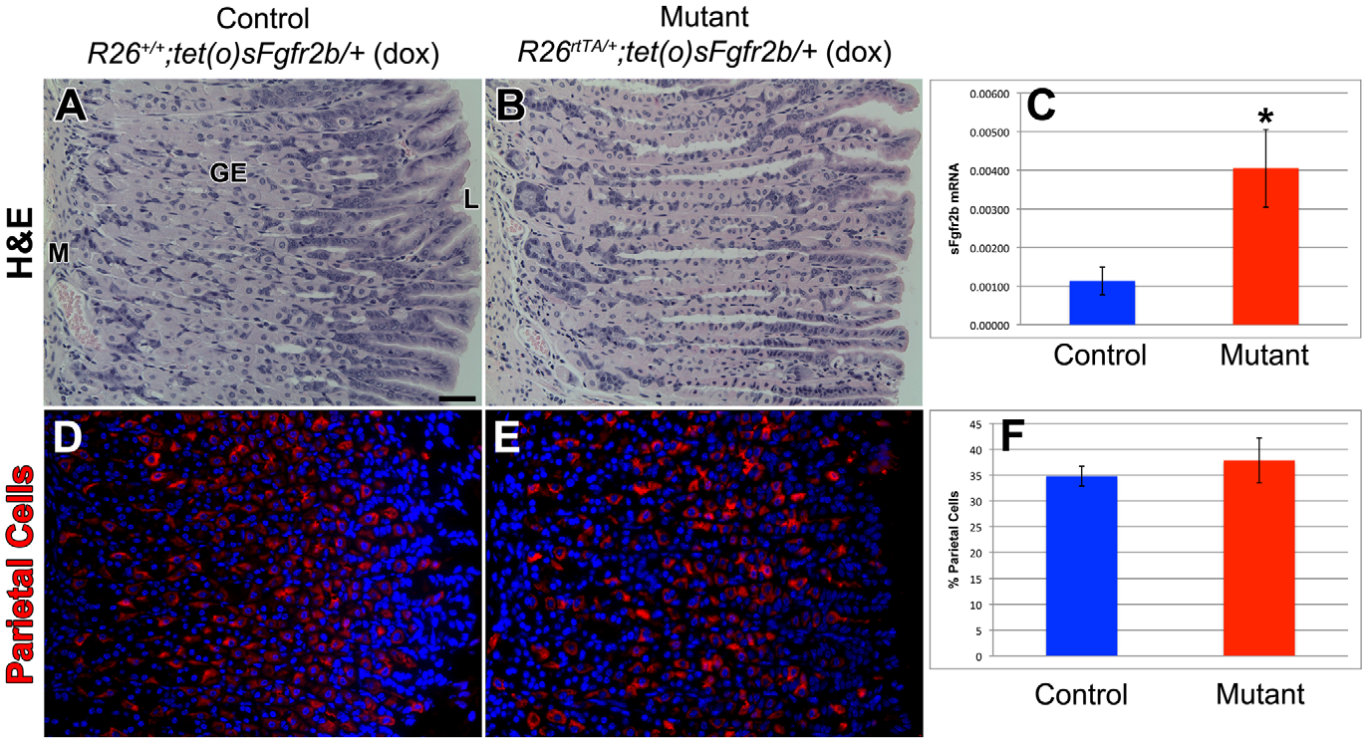
FGF10-FGFR2b mediated signaling is not required for parietal cell differentiation during homeostasis. Hematoxylin and eosin staining of sections of adult glandular stomach in control littermate (*R26^+/+^; tet(O)sFgfr2b/+* with dox) (A) and mutant mice (*R26^rtTA/+^; tet(O)sFgfr2b/+* with dox) (B) showing normal stomach histology. (C) qRT-PCR confirming a significant increase in the expression of *sFgfr2b* in the mutants as compared to controls (*p<0.05). Parietal cells are identified by H/K ATPase immunostaining of sections of adult glandular stomach in control littermate (*R26^+/+^; tet(O)sFgfr2b/+* with dox) (D) and mutant mice (*R26^rtTA/+^; tet(O)sFgfr2b/+* with dox) (E) and (F) quantification reveals no significant change in parietal cell number. Dox was given for 3 months. The scale bar is 50 µm. M = mesenchyme, GE = gastric epithelium, L = lumen.

Since the lifespan of a parietal cell is 54 days [37], a longer-term study was required to confirm the dispensability of FGF10-FGFR2b mediated signaling for parietal cell differentiation during adult glandular stomach homeostasis. In order to accomplish this, we induced ubiquitous overexpression of a dominant-negative *sFgfr2b* by feeding doxycycline to adult (4 week old) mutant mice and control littermates for 3 months prior to sacrifice. The expression of *sFgfr2b* in the mutant mice was significantly higher than controls as shown by qRT-PCR (Figure 7C). Hematoxylin and eosin staining of sections of control littermate (Figure 7A) and mutant (Figure 7B) adult glandular stomachs demonstrated normal histology with visible parietal cells. There was no difference in the percentage of parietal cells in the gastric epithelium of the mutants (Figure 7E) compared to controls (Figure 7D) as evidenced by H/K ATPase immunostaining (37.9±4.4% vs. 34.8±1.9%, p-value = 0.277, n = 3 for each genotype) (Figure 7F). These results confirm that FGF10-FGFR2b mediated signaling is not required for parietal cell differentiation during adult glandular stomach homeostasis.

## Discussion

FGF10-FGFR2b mediated signaling is essential for embryonic gastric development [11], [13], [23]. However, little is known about the role of FGF10-FGFR2b signaling during maintenance of mature gastric epithelium. We sought to understand FGF10-FGFR2b mediated signaling during adult glandular stomach homeostasis. Double transgenic mice that ubiquitously overexpress *Fgf10*, displayed increased epithelial proliferation, and considerably altered differentiation of three of the four epithelial cell lineages in the stomach. However, double transgenic mice overexpressing a soluble form of Fgfr2b, FGF10's main receptor, revealed that FGF10-FGFR2b signaling is not necessary for epithelial proliferation and differentiation.

We found that *Fgf10*, its receptors, *Fgfr1b* and *Fgfr2b*, and most of the other FGFR2b ligands (*Fgf-1, −7, −22*), were present in the adult stomach. These results are supported by the expression of these genes during gastric organogenesis in the mouse [11] and chick embryo [12], [23]. Despite some differences in expression between the mouse and chick embryo, the gastric expression of *Fgf10* and its main receptor *Fgfr2b* remains conserved between species and both are present during development and homeostasis. The principal receptor for FGF10 is FGFR2b [38], [39] and inactivation of *Fgf10* or *Fgfr2b* in mouse embryos leads to remarkably similar phenotypes [34], [40], whereas FGF10 binds FGFR1b with a lower affinity [41]. However, the presence of both *Fgfr1b* and *Fgfr2b*, as well as some of the FGFR2b ligands (*Fgf-1, −7, −10,* and *−22)* in adult stomach may allow for some intrinsic redundancy in FGF signaling. Certainly the expression of *Fgf10* and its receptor, *Fgfr2b*, in adult stomach suggests that FGF10-FGFR2b signaling occurs postnatally.

Multiple studies have recognized FGF10 as a promoter of epithelial proliferation during tracheal [42], colonic [19], [20], and gastric [13], [23] development as well as postnatally during mammary gland [36] and incisor [35] homeostasis. Many of these characterize a loss of function approach, demonstrating decreased epithelial proliferation in *Fgf10^−/−^* [13], [20], [42] and/or *Fgfr2b* ^−/−^ [13], [19] mice as well as in transgenic mice that overexpress soluble *Fgfr2b* [35], [36]. Only two previous studies report a gain of function approach similar to ours, investigating the effects of *Fgf10* overexpression during gastric organogenesis, with contradictory results [11], [23]. Shin et al. demonstrated a modest increase in glandular epithelial proliferation in chick embryonic stomach with viral-mediated overexpression of *Fgf10* compared to uninfected controls [23]. Our results align with these results in organogenesis although we are studying a much later time point when we demonstrate that FGF10 has a mitogenic effect during adult stomach homeostasis.

In addition, FGF10 plays an important role in epithelial differentiation during the development of numerous organs [13], [42], [43], [44]. In particular, during gastric organogenesis, the loss of FGF10-FGFR2b mediated signaling results in the complete absence of parietal cells [13]. This indicates that FGF10-FGFR2b mediated signaling is essential for parietal cell differentiation during stomach development, and yet, *Fgf10* overexpression during this same time period also has a negative effect on parietal cells with a 78% reduction at E18.5 [11]. Interestingly, we found that FGF10-FGFR2b mediated signaling is not required for parietal cell differentiation during adult stomach homeostasis, but *Fgf10* overexpression caused a similar reduction in the parietal cell lineage as occurs during organogenesis. Thus, FGF10 at high levels down-regulates parietal cell differentiation during both stomach development and homeostasis, but once gastric organogenesis is complete, FGF10-FGFR2b mediated signaling appears to be dispensable for parietal cell differentiation.

Endocrine cells represent a small, but heterogeneous population of cells that secrete a variety of hormones in the gastric epithelium. Terminal endocrine cell fate appears unaffected by the loss of FGF10-FGFR2b mediated signaling during development [13]; however, ectopic *Fgf10* overexpression results in significant suppression of the endocrine cell lineage [11]. Unlike these developmental studies, we did not observe any change in endocrine cell differentiation upon overexpression of either *Fgf10* or soluble *Fgfr2b* during stomach homeostasis.

FGF10-FGFR2b mediated signaling promotes chief cell differentiation during gastric organogenesis as evidenced by the reduced abundance of chief cells in *Fgf10^−/−^* and *Fgfr2b^−/−^* E18.5 stomachs [13] and the increase in chief cells in stomachs with ectopic *Fgf10* overexpression [11], In contrast, we observed a significant decrease in chief cells upon *Fgf10* overexpression in adult glandular stomach homeostasis. We identified chief cells by immunostaining for intrinsic factor and these previous studies stained for pepsinogen [11], [13], but both are well-established markers of chief cells in mice [21], [28], [29], [45] and variation in immunohistochemical staining alone seems unlikely to account for this observation. It is possible that during homeostasis *Fgf10* levels have adverse effects on chief cells as opposed to developmental stages.

It is known that FGF10-FGFR2b mediated signaling is not required for mucous cell differentiation during stomach development [13], and we found the same to be true during homeostasis. However, FGF10 has been shown in previous studies to either induce mucus-secreting cell number [24] or to shift cell location within the gastric gland from the lumen towards the gland base [11], [23]. We observed both phenomena during adult glandular stomach homeostasis. This phenotype, combined with the loss of parietal cells, is often described as “antralization” and is observed in gastric metaplasias, such as IM and/or SPEM. The loss of parietal cells usually results in metaplasia [4], [32], [33]. During this process, chief cells transdifferentiate by losing expression of MIST1 and gaining expression of CDX2 in IM or TFF2 in SPEM [21], [33]. Perhaps the reduction of chief cell number in our model is due to transdifferentiation, however, this would require further investigation to confirm.

We are not the first to observe a SPEM-like phenotype in association with FGF10 signaling. Spencer-Dene et al. demonstrated a disproportionately underdeveloped antrum with an enlarged simple unbranched gastric epithelium for both *Fgf10^−/−^* and *Fgfr2b^−/−^* embryos indicating a role of FGF10-FGFR2b mediated signaling in promoting antralization [13]. Furthermore, overexpression of *Fgf10* during stomach development resulted in a SPEM-like phenotype with a shift in localization of TFF2 mRNA in the mouse and cSP mRNA in the chick, in addition to a reduction in the number of parietal cells in the mouse [11] [23]. cSP is a marker of luminal epithelial cells in the chick, the analog to mucous neck cells in the mouse [8]. Nyeng et al. speculated that the antralization of the corpus in their model could be explained by increased FGF10 availability in the corpus as compared to the normal gradient of *Fgf10* expression, which is higher in the antrum and lower in the corpus [11]. This could explain the SPEM-like phenotype seen during homeostasis as well, since *Fgf10* was ubiquitously overexpressed in our model. Despite *Fgf10* overexpression resulting in a SPEM-like phenotype, well-established markers failed to confirm metaplasia during homeostasis, as has been similarly reported during stomach development [11]. Hence, we cannot exclude the possibility that FGF10-FGFR2b signaling may play a role in gastric metaplasia. In fact, ETV4, a downstream target of FGF10, is upregulated in human gastroadenocarcinoma samples and is associated with decreased survival [46], [47]. Since SPEM often results from parietal cell loss, and the average parietal cell lifespan is approximately 54 days [37], only 10 days of *Fgf10* overexpression may not induce sufficient parietal cell loss and consequent metaplasia. Perhaps a higher *Fgf10* expression level and/or a longer exposure time are required for the detection of metaplasia markers in our homeostasis model. When mice are infected with *Helicobacter felis*, SPEM does not develop for 4–6 months [4]. Unfortunately, we were unable to overexpress *Fgf10* for more than 10 days since the mice develop systemic toxicity. More investigation is needed to confirm the link between FGF10-FGFR2b mediated signaling and metaplasia. The use of gastric-specific overexpression could be considered for future studies, to prolong the exposure.

In conclusion, these results demonstrate, for the first time, the role of FGF10-FGFR2b mediated signaling during adult glandular stomach homeostasis. *Fgf10* overexpression affects both gastric epithelial proliferation and differentiation. FGF10 promotes mucous neck cell differentiation while suppressing parietal and chief cell differentiation, producing a SPEM-like phenotype; but does not appear to induce metaplasia. In spite of the evidence for a role for FGF10-FGFR2b mediated signaling during stomach homeostasis, FGFR2b signaling inhibition did not impair gastric epithelial proliferation or differentiation. This suggests that FGF10 may act through another receptor such as FGFR1b during stomach homeostasis. Thus, FGF10-FGFR2b mediated signaling is not required during adult glandular stomach homeostasis.

## Materials and Methods

### Ethics Statement

All experimental protocols were in accordance with the recommendations in the Guide for the Care and Use of Laboratory Animals of the National Institutes of Health. The protocol was approved by the Institutional Animal Care and Use Committee at Children's Hospital Los Angeles (IACUC protocol #193). All mice included in this study were humanely euthanized by exposure to CO_2_ in an inhalation chamber.

### Mice

All animals were housed in the Saban Animal Care Facility of the Saban Research Institute at Children's Hospital Los Angeles, Los Angeles, California. Animals were maintained in a temperature-regulated environment on a 12-hour light-dark cycle and given access to chow and water *ad libitum*. Wild type mice were C57Bl/6 mice from the Jackson Laboratory.

### PCR

RNA was isolated from adult wild type (C57Bl/6) mouse glandular stomach (n = 3) and E14.5 wild type whole mouse embryo (n = 2) using the Qiagen RNA extraction kit (Qiagen, Valencia, CA). The iScript^TM^ cDNA Synthesis Kit (BioRad, Hercules, CA) was used to synthesize cDNA. All PCR reactions were performed following the manufacturer's instructions using either the Taq PCR Master Mix (Qiagen, Valencia, CA) or 2x MyTaq Red mix (Bioline, Tauton, MA) in a C1000^TM^ Thermal Cycler (BioRad, Hercules, CA). Primers were designed specifically to span an intron using the National Center for Biotechnology Information (NCBI) website (http://www.ncbi.nlm.nih.gov/). The following primer sequences were used: (forward 5′-3′, reverse 5′-3′): *Fgfr1b* CTTGACGTCGTGGAACGATCT, CCACAGGTCTGGTGACAGTGA; *Fgfr2b* AACGGGAAGGAGGTTTAAGCAG, GGACAGTGAGCCAGGCAG; *Fgf1* ACCTTCGCAGCCCTGACCGA, GACCGCGCTTACAGCTCCCG; *Fgf3* CCATGAACAAGAGAGGACGGCTGTATG, TCTGCAGCAGCCGCACCATCTC; *Fgf7* TCTGCTCTACAGATCATGC, TAGGTTATTGCCATAGGAA; *Fgf10* CACATTGTGCCTCAGCCTTTCC, CCTGCCATTGTGCTGCCAGTTAA; *Fgf20* GGGACCTCACAGGAGTAACCCA, TGCGACCCGTGTCTCCATGT; *Fgf22* CGCATCTGGAGGGCGACGTG, CCACAGAGTAGACCCGCGACCC.

### Analysis of LacZ expression

*Fgf10^Mlc-nlacZ-v24^* mice [48] (hereafter called *Fgf10^lacZ^*) were maintained on a mixed agouti background. Samples were briefly fixed in 4% PFA. *lacZ* expression on *Fgf10^lacZ/+^* (n = 3) and wild type *Fgf10^+/+^* mice (n = 3) adult glandular stomach were monitored by β-galactosidase activity using 1mg X-gal/ml dimethylformamide in 5 mM K3Fe(CN)6/5 mM K4Fe(CN)6/2 mM MgCl2 in D-PBS (pH 7.4) as previously described [49]. These samples were then paraffin-embedded, sectioned at 5 μm intervals, and mounted on slides. Slides were deparaffinized, counter-stained with nuclear fast red, dehydrated, cleared with histochoice, and mounted with Permount (Fisher Scientific, Fair Lawn, New Jersey).

### Generation of R26^rtTA^; tet(O)Fgf10 and R26^rtTA^; tet(O)sFgfr2b mice

In order to generate mice that express rtTA under the ubiquitous Rosa26 (R26) promoter, *CMV^Cre^* mice [50] were mated with *R26^rtTA^* mice [51]. The *tet(O)Fgf10* responder line [52] was then crossed with this constitutive rtTA mouse line to generate double transgenic heterozygous animals (*R26^rtTA/+^; tet(O)Fgf10/+*). These mice ubiquitously overexpress *Fgf10* when induced with doxycycline. The constitutive rtTA mouse line was also crossed with the *tet(O) soluble Fgfr2b* responder line [53] to generate double transgenic heterozygous animals (*R26^rtTA/+^; tet(O)sFgfr2b/+*), which ubiquitously express the dominant-negative *sFgfr2b* [54] as previously described and validated [35], [36]. All mice were generated on a CD1 mixed background and genotyped as previously described [50], [51], [52], [53]. Inducible ubiquitous overexpression of *Fgf10* or *sFgfr2b* in mutant mice was achieved by administration of doxycycline-containing food (Rodent diet with 0.0625% Doxycycline, Harlan Teklad TD01306) (hereafter called dox).

For the overexpression of *Fgf10* experiments, double transgenic mutant adult (4 week-old) mice (*R26^rtTA/+^; tet(O)Fgf10/+)* were given dox for a period of 10 days. Double transgenic littermates (*R26^rtTA/+^; tet(O)Fgf10/+)* without dox were used as controls. A total of three mutants and three controls were used to investigate histology, epithelial proliferation and differentiation, and metaplasia.

Regarding the overexpression of *sFgfr2b*, double transgenic mutant adult (4 week-old) mice (*R26^rtTA/+^; tet(O)sFgfr2b/+)* were given dox for a period of 1 month or 3 months. Single transgenic littermates (*R26^+/+^; tet(O)sFgfr2b/+)* with dox were used as controls. A total of three mutants and three controls were used to investigate histology, epithelial proliferation and differentiation for each time period.

### Tissue collection

Adult glandular stomach, from control and mutant mice, were collected at the conclusion of dox administration (10 days for *Fgf10* overexpression (n = 5 controls, n = 5 mutants) and 1 month (n = 5 controls, n = 5 mutants) and 3 months (n = 3 controls, n = 3 mutants) for *sFgfr2b* overexpression). Approximately 30 mg of tissue was saved in RNA*later®* (Sigma-Aldrich, St. Louis, MO) for qRT-PCR. The remaining tissue was fixed in 10% formalin and embedded in paraffin. Adult glandular stomach from wild type (C57Bl/6) mice (n = 3) was also fixed in 10% formalin and embedded in paraffin. All paraffin embedded tissue was serially sectioned at 5 μm intervals and mounted on slides for staining analysis.

### RNA extraction and Quantitative RT-PCR (qRT-PCR)

RNA was isolated from the glandular stomachs of control and mutant mice using a Qiagen RNAeasy kit (Qiagen, Valencia, CA) in accordance with the manufacturer's directions. A Nano Drop ND-1000 was used to determine the concentration of the purified RNA.

RNA (1 μg) was reverse-transcribed using the iScript^TM^ cDNA Synthesis Kit (BioRad, Hercules, CA) in accordance with the manufacturer's directions. 2ul cDNA were used for each of the qRT-PCR reactions using the primers and probes designed by the online Roche software: Probe finder version 2.20, https://www.roche-applied%1Escience.com/sis/rtpcr/upl/adc.jsp. The following primer and corresponding probes were used (forward 5′-3′, reverse; 5′-3′, probe #): *Fgf10* CGGGACCAAGAATGAAGACT, AACAACTCCGATTTCCACTGA, probe 80; *sFgfr2b* GAAGGAGATCACGGCTTCC, AGACAGATGATACTTCTGGGACTGT, probe 108. All qRT-PCR reactions were performed with Roche FastStart TaqMan® Probe Master kits, according to the manufacturer's instructions in a Roche Light Cycler 1.5 Real-Time PCR machine. Each reaction was run in triplicate and expression levels of genes of interest were normalized to levels of *Gapdh.*

### Immunofluoresence staining

Slides were deparaffinized and rehydrated through a successive ethanol concentration to water. An antigen retrieval step was performed by boiling the slides in a microwave for 12 min in 10 mM sodium-citrate buffer pH = 6.0 (PCNA, GSII, CGA, IF, H/K ATPase, HE4). Slides were blocked with universal blocking solution with 2% goat or donkey serum for 30 minutes, followed by incubation with the primary antibody diluted in universal blocking solution with 2% goat or donkey serum overnight at 4°C. Primary antibodies included: mouse anti-proliferating cell nuclear antigen (PCNA) (1∶100, Vector Laboratories), rabbit anti-chromogranin A (CGA) (1∶100, ABcam), goat anti-intrinsic factor (IF) (1∶2000, a gift from Jason Mills, Washington University, St. Louis, MO), mouse anti-H/K ATPase β (H/K ATPase) (1∶100, ABcam), and rabbit anti-HE4 (1∶50, ABcam). Slides were then incubated with secondary antibody diluted in universal blocking solution for one hour at room temperature. Secondary antibodies included: Cy3 conjugated goat anti-rabbit or mouse (1∶200), Cy3 conjugated donkey anti-goat (1∶200), or lectin GS-II from *Griffonia simplicifolia*, Alexa Fluor® 488 Conjugate (GS-II) (1∶2000, Invitrogen). Slides were mounted using Vectashield with DAPI (Vector Laboratories). Images were acquired using a color camera attached to an upright fluorescent microscope (Leica DM5500).

### Immunohistochemistry

Slides were deparaffinized and rehydrated through a successive ethanol concentration to water. An antigen retrieval step was performed by boiling the slides in a microwave for 12 min in either Tris-EDTA (10 mM Tris Base, 1mM EDTA Solution, 0.05% Tween 20, pH = 9.0) (FGFR1 and FGFR2) or 10 mM sodium-citrate buffer pH = 6.0 (CDX2). Slides were then incubated with 15% H­_2_O_2_ in 1x PBS for 15 minutes. Then slides were blocked with universal blocking solution with 2% goat serum for 30 minutes, followed by incubation with the primary antibody diluted in universal blocking solution with 2% goat serum overnight at 4°C. Primary antibodies used included: rabbit anti-FGFR1 (1∶100, Flg (C-15) Santa Cruz), rabbit anti-FGFR2 (1∶100, Bek (C-17) Santa Cruz) and rabbit anti-CDX2 (1∶50, BioGenex, Fremont, CA). The Envision kit (Dako) was used to reveal the slides. Slides were incubated with labeled polymer-HRP for 30 minutes at room temperature. Slides were then revealed using DAB+ chromogen in substrate buffer for 5 minutes. Slides were then counter-stained with hematoxylin, dehydrated to 100% EtOH, cleared with xylene, and mounted with Permount (Fisher Scientific, Fair Lawn, New Jersey). Images were acquired using a color camera attached to an upright fluorescent microscope (Leica DM5500).

### Cell counting and quantification

Epithelial nuclei, PCNA-positive and H/K ATPase-positive stained cells were counted semi-automatically using MetaMorph 7.7.3.0 software (Molecular Devices, Sunnyvale, CA). First, the blue (DAPI) channel was processed as follows: background was subtracted by manual thresholding, intensity was normalized by dividing by a 20×20 low-pass filtered copy of the channel, and the normalized nuclei were smoothed with a 5×5 median filter to preserve edges. A binary image of the smoothed nuclei was created by thresholding automatically using the isodata histogram algorithm under visual inspection to manually adjust the threshold value if needed. Overlapping binary nuclei were separated by Watershed segmentation using FoveaPro 3.0 software (Reindeer Graphics, Asheville, NC). To count H/K ATPase-positive cells, the red (Cy3) channel was processed in the same manner as the blue channel except that a legacy heuristic algorithm was more appropriate for thresholding and an additional step to fill all dark holes was added just prior to Watershed segmentation to fill the unstained nuclei and prevent over-segmentation. To count PCNA-positive cells, the red (Cy3) channel was pre-processed in the same manner as for H/K ATPase-positive cells, except that the median filter kernel was 3×3. Binary objects were counted with the Integrated Morphometry Analysis function of MetaMorph. Object filters were set to exclude staining and processing artifacts from the counts, as well as mesenchymal nuclei: elliptical form factor (ratio of length to breadth) ≥3.0 and area ≥100 pixels for epithelial nuclei, ≥200 pixels for H/K ATPase-positive cells, and ≥40 pixels for PCNA-positive cells. GSII, CGA and IF-positive stained cells were counted by hand using ImageJ software (http://rsbweb.nih.gov/ij/).

The total number of PCNA, GSII, CGA, IF, and H/K ATPase positive cells and the total number of epithelial cells were counted separately for 3 randomly selected high power fields (HPF = 20x magnification) in the corpus of a single mouse and averaged. Specifically, five mutant and five control mice were counted for all *Fgf10* overexpression, and for *sFgfr2b* overexpression induced for one month. For the *sFgfr2b* overexpression induced for three months, we analyzed three mutants and three controls. The total number of cell-type positive cells per the total number of epithelial cells, or percent cell-type positive was calculated for each sample.

### Statistical Analysis

Data are presented as the mean±standard error of the mean (SEM). Statistical analysis was performed using a one-tailed two-sample equal variance (homoscedastic) Student's *t*-test (Excel, Microsoft). P-values <0.05 were considered significant.
